# Effect of Sterilization Methods on Chemical and Physical-Mechanical Properties of Cotton Compresses

**DOI:** 10.3390/molecules29153541

**Published:** 2024-07-27

**Authors:** Maja Somogyi Škoc, Jana Juran, Iva Rezić

**Affiliations:** 1 Department of Materials, Fibres and Textile Testing, Faculty of Textile Technology, University of Zagreb, 10000 Zagreb, Croatia; juran.jana94@gmail.com; 2 Department of Applied Chemistry, Faculty of Textile Technology, University of Zagreb, 10000 Zagreb, Croatia; iva.rezic@ttf.unizg.hr

**Keywords:** medical textiles, sterilization, steam sterilization, sterilization with ethylene oxide, cotton compresses, textile testing, damage to textiles

## Abstract

The aim of this work was to determine the changes in the chemical and physical-mechanical properties of gauze compresses under the influence of various sterilizations. Gauze compresses are made of cotton; therefore, all methods used focused on cotton. The methods used to test possible damage to cotton materials (pH value (pH paper, KI starch paper), yellowing test, Fehling reaction, reaction to the formation of Turnbull blue (Berlin blue), microscopic staining with methylene blue and swelling reaction with Na-zincate) did not show that the sterilizations affected the cotton compresses. The morphological characteristics were examined with a scanning electron microscope (SEM). The SEM images showed that there were no morphological changes in the cotton fibers. FTIR-ATR spectroscopy revealed that the sterilization processes did not alter the characteristic bands of the cotton. The length of the macromolecules was increased (DP), showing that the sterilization processes had affected the cotton. The results of the wet strength test followed. The samples showed values below 100%, with the exception of two samples. It is known from theory that the relative wet strength is less than 100% when the material is damaged. The *t*-test performed on the strength results showed that the *p*-value was greater than 0.05 for all samples tested, with the exception of one sample. The degree of swelling capacity was determined, with non-sterilized samples having the highest capacity, followed by samples sterilized with ethylene oxide and then samples sterilized by steam sterilization. The results obtained are a contribution to the innovation of the topic of this work and a scientific confirmation for manufacturers and anyone interested in the influence of the sterilization process on natural fibers (cotton).

## 1. Introduction

Cotton gauze has a long tradition as an effective medical fabric for compressible and non-compressible wounds. It is recognized primarily for its safety, natural softness, non-allergenic properties, breathability, stability, blood absorbency and ease of application [1]. It remains the most commonly used hemostasis for the control of traumatic bleeding. Cotton gauze has the ability of a hemostatic mechanism. This mechanism is activated by the contact of platelets with the cotton fiber. Upon this contact, the cotton fiber has the ability to absorb blood fluid quickly and causes blood cells and platelets to combine to form blood clots [2]. Cotton gauze or gauze is often used as a generic term for a wide range of compresses and dressing products. The two main groups are woven and non-woven gauze. Woven products, often referred to as absorbent gauze, are generally made from 100% natural cotton yarns and have been manufactured in the same way for centuries. In woven cotton gauze, horizontal warp threads are twisted around vertical weft threads to create a loose, interlocking pattern. This technique also results in the cotton gauze having a soft and crinkled texture. Non-woven gauze compresses are generally made from viscose or synthetic fiber blends [3]. As non-active medical devices, gauze compresses must be made from pure, absorbent cotton gauze that complies with the EN 14,079 standard and the European Pharmacopoeia [4,5]. The compress is stacked in layers, and the edges are folded so that no threads come out. Compresses are produced in various sizes and packaging in sterile or non-sterile form. It is very important that cotton compresses contain at least one interwoven strip at the edges so that the thread is not pulled out. The compresses contain a minimum of eight and a maximum of 16 flat cotton gauzes folded at certain angles [6]. According to the European Union Regulation 2017/745, compresses are medical devices [7]. In brief, the regulation defines a medical device as an instrument, apparatus, implant, reagent, material or other article intended by the manufacturer to be used alone or in combination with others for one or more specified medical purposes, for example, for the treatment or alleviation of an injury in humans, and which does not exert its main effect by pharmacological, immunological or metabolic means.

Compresses must be sterilized to clean and protect superficial wounds, sutured wounds, post-operative wounds, etc. Today, there are various methods of sterilizing medical devices, for which general criteria for sterility have been established [8]. For medical devices, the requirements for the development, validation and routine control of a sterilization process are standardized. The sterilization of compresses by heat sterilization is performed according to ISO 11737-2 [9], and the sterilization of compresses with ethylene oxide according to ISO 11135 [10]. In order to find out whether sterilization affects the cotton fibers, various methods were therefore used, some of which are used as methods for determining damage to cotton fibers. In order to find out whether sterilization has an effect on the cotton fibers of the compresses, the physical and mechanical properties were observed in our preliminary work. It was found that sterilization by steam sterilization showed a negligible increase in the degree of polymerization and slightly reduced maximum strength in the dry state [11]. When searching the available literature for the keywords compresses, cotton, textile, sterilization (steam, ethylene oxide), impact, damage to cotton fiber, etc., no articles were found apart from the author’s preliminary work [11]. Among the available articles for the above keywords, no articles were found that were similar or approximately similar to this article. Articles on the topic of sterilization from 1961 to 2023 were found. One article investigated the factors influencing the efficient steam sterilization of dressings [12], while another article evaluated the effectiveness of a 2 × 1 serge cotton fabric as a microbial barrier after several washing and steam sterilization processes [13]. One article investigated the potential of using 2450 MHz microwave radiation to dry and sterilize polyester and cotton fabrics against Staphylococcus aureus, Escherichia coli and Bacillus cereus [14]. The aim of this article was to test the effectiveness of cotton fabric made of 2 × 1 serge as a microbial barrier when new and after repeated washing and steam sterilization [15]. The results and practical solutions chosen to perform a sterilization validation for compresses according to ISO 11137-1: 2006 were also presented [16,17]. The sterilization effects of low-pressure plasmas on cotton fabrics inoculated with bacteria were investigated, and the influence of the plasma treatment on the physical properties of the fabrics was tested. It was found that the plasma treatment had no effect on the final tensile strength and surface morphology of the fabrics, as a relatively low plasma temperature was utilized [18]. The influence of washing and steam sterilization on the properties of polyurethane-coated fabrics [19] and the sterilization efficiency of cotton contaminated with pathogens in a washing machine [20] were investigated. Gamma radiation for the sterilization of textile materials for personal protective equipment was also investigated [21]. A research paper provided fundamental insights into the synthesis of NABPE and the prolonged biocidal efficacy of M-cotton/NABPE, paving the way for an economical and environmentally friendly antibacterial coating suitable for the finishing of cotton fabrics [22]. In one article, the authors evaluated the effects of common disinfectants, sterilizers and DNA degrading agents on cotton textiles to assess the possibility of identifying the type of agent used based on the changes observed on the treated textiles and fibers [23], etc.

The aim of this work was to determine the changes in the chemical and physical-mechanical properties of gauze compresses under the influence of various sterilizations. To the best of our knowledge, based on the available data, the topic covered in this paper has not yet been researched and can be considered innovative.

**Figure 1:**
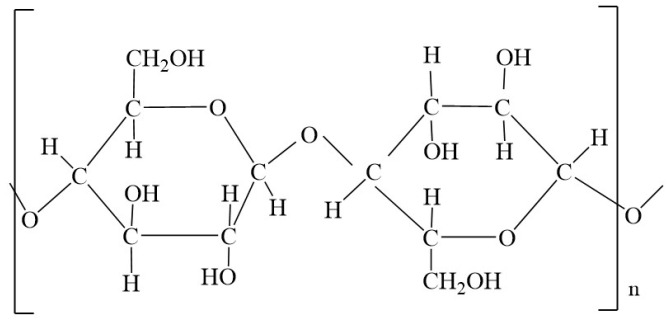
The structural formula of the macromolecule cellulose contains cellobiose as the basic structural unit, i.e., a molecule made up of two glucose residues.

To determine whether sterilization has an effect on the properties of cotton fibers of compresses, a variety of methods to determine the damage to cotton fibers (chemical methods and physical-mechanical methods) were performed in this work. Most damage to cotton materials is due to the chemical effects of various substances. These can be acids, salts, strong alkalis (especially at high temperatures and in the presence of atmospheric oxygen), oxidizing agents and, in some cases, reducing agents [24]. The effects of heat and strong light as well as mold, bacteria, insects and mechanical influences can also cause damage. The cellulose molecule is a long-chain molecule whose members are β-glucose units (Figure 1). The number of basic units is expressed by the degree of polymerization (DP), which is an average value. For natural cellulose, DP is 7000–14,000, and for regenerated cellulose, DP is up to 1000 [25].

**Figure 2:**
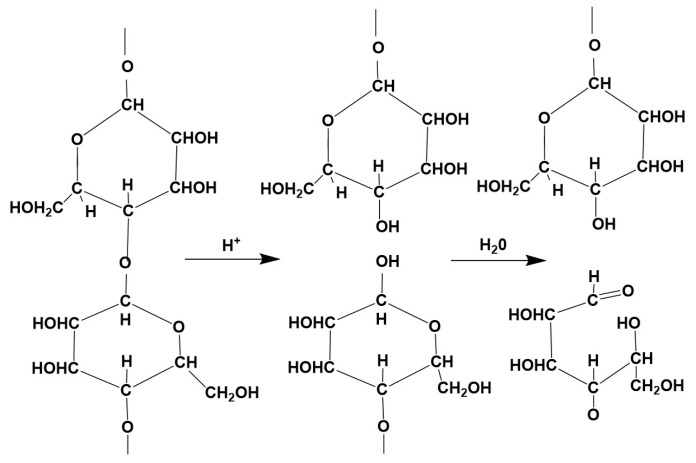
Hydrocellulose.

Any chemical damage causes the cellulose to break down; the long-chain molecules are cleaved at 1.4 bonds, which reduces the DP and at the same time leads to a decrease in breaking strength. If the damage is caused by the action of acids (hydrolytic decomposition), the reducing power of the cellulose increases, as aldehyde groups are formed at each cleavage site—including at the C_1_ atom—which has a strong reducing effect, so that Cu_2_O can be extracted from the Fehling solution, for example [26]. This was used to quantitatively determine the copper number. Cellulose decomposed by acid is called hydrocellulose (Figure 2).

**Figure 3:**
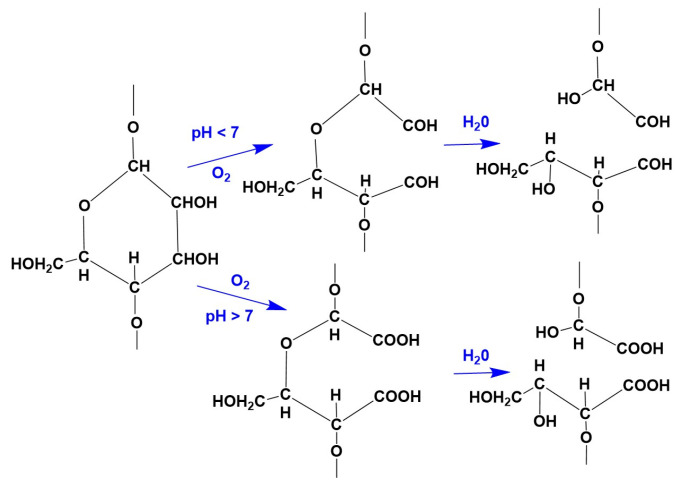
Oxycellulose.

If the damage is caused by the cleavage of the 1.4-bond, the aldehyde group formed at the C_1_ atom can also, depending on the conditions (temperature, concentration, pH value), be quickly and completely oxidized to the carboxyl group, which no longer has a reducing effect but has the ability to form salt-like compounds that can bind basic dyes such as methylene blue, malachite green, etc., in increased quantities. The oxidation of alcohol groups (on C_6_, C_3_ and C_2_ atoms) does not take place under the conditions of the textile finishing process [26]. Cellulose damaged by oxidation is called oxycellulose (Figure 3).

In general, hydrocellulose is a more or less acid-degraded cellulose containing aldehyde groups. Compared to undamaged cellulose, it has a lower DP, a lower breaking strength and a higher copper content. Oxycellulose is cellulose damaged by oxidizing agents and contains mainly carboxyl groups and fewer aldehyde groups. It also has a lower DP than intact cellulose, a lower strength and a higher copper number [26]. The strength test can be used to test for damage. In the instrumental determination of strength, it is possible to detect damage of no less than 7 to 10%, while chemical methods are somewhat more accurate and allow the detection of damage of 2 to 3% [25]. If it is determined that the sample is damaged, the first step is to examine whether the agent that caused the damage is still present on the material. By applying the following test methods, the type and intensity of damage to the cotton material can be determined: indicator reaction (pH paper, KI starch paper), pH value of the aqueous extract, yellowing test, Fehling reaction, Turnbull’s blue staining reaction (Berlin blue), microscopic staining with methylene blue and swelling reaction with Na-zincate [26].

## 2. Materials and Methods

### 2.1. Preparation of Compresses

Compresses were used for surgery and post-operative wound treatment. To determine the changes in the chemical and physical-mechanical properties of gauze compresses under the influence of different sterilizations, compresses with two different batch numbers were used (compress 1 and compress 2). The composition of the compresses was determined according to EN 14079:2008 [4]. The mass per unit area was determined in accordance with EN 14079:2008 [4]. The thickness was determined according to ISO 5084 [27].

### 2.2. Methods of Sterilizing

The compresses were sterilized by steam sterilization and ethylene oxide sterilization. The manufacturer has carried out both sterilization procedures mentioned in order to obtain all the conditions to which the compresses are normally exposed during sterilization and to achieve the most realistic and meaningful results possible in this work. As trade secrets, the four parameters of steam sterilization—steam, pressure, temperature and time—were not specified by the manufacturer. A temperature of 121–134 °C, a duration of 3–15 min and a pressure of 1.2–2.5 bar were presumed to have been used by the manufacturer [28]. The following sterilization steps were carried out during steam sterilization of the compresses: preparation: placing the object in the steam sterilizer, removing residual air, heating and sterilizing, cooling, and drying. Steam sterilization was selected as a non-toxic, inexpensive, fast microbicidal, sporicidal, fast heating and penetrating method for tissue (compresses). Sterilization was carried out at the end of compress production as a discontinuous process in an autoclave. The sterility of the compresses was verified by sterility tests in accordance with EN ISO 11737-2 [9]. 

The parameters of ethylene oxide sterilization were defined by the manufacturer, and were also trade secrets. Ethylene oxide sterilization (ETO) uses a combination of vacuum, humidity, temperature and gas to sterilize materials at lower temperatures. A temperature of 35–60 °C, a humidity of 30–90%, a temperature of the ventilation system of 37–50 °C and a concentration of 400–650 mL ethylene oxide were presumed to have been used by the manufacturer [29]. A typical ETO sterilization cycle consists of at least three phases: preconditioning, sterilization, and aeration (degassing). The duration of the cycle is normally more than 14 h. The manufacturer, in accordance with ISO 11135, tested sterilization with ethylene oxide [10]. For the sterilization of long-lumen instruments and all materials that need to be sterilized but cannot tolerate higher temperatures, this method is commonly used. 

### 2.3. Test Method for pH of the Water Extract from Wet-Treated Textiles

The pH value of the water extract from wet-treated textiles was determined according to AATCC test method 81-1988 [30]. Distilled water (250 mL) was boiled for 10 min. The sample (10 g) was immersed and boiled for a further 20 min. The closed beaker and the contents were cooled to room temperature. The pH value was determined using a pH meter CG 842, Schott, Mainz, Germany. The pH of the water extract depends on the chemical treatment with which the compresses were previously treated. It is an excellent method to test the effect of sterilization on cotton. 

### 2.4. Determination of the Degree of Polymerization

The degree of polymerization was determined using the Ubbelohde viscometer, Schott, Mainz, Germany with suspended level for determining the absolute and relative kinematic viscosity of liquids with Newtonian flow behavior. The calibrated viscometer has a manufacturer’s certificate in accordance with DIN 55 350, Part 18 [31]. The degree of polymerization (DP) was measured according to DIN 54270-2-1977-08 and calculated according to the Schultz–Blanschke equation [32]. The Ubbelohde viscometer consists of three tubes. Through one of them, which remains connected to the atmosphere, an air bubble enters the reservoir, causing the liquid to flow continuously along the wall and flow out under constant conditions. 

Copper-ethylene diamine (Cuen) is used as a solvent. The mass of the sample must be adjusted according to the degree of polymerization, i.e., for cotton with a DP of more than 1500, the weight of the cotton fibers must be 0.02 g. The crushed fiber sample was placed in a 50 mL volumetric flask. The volumetric flask was then filled up to the mark with solvent, tightly closed and shaken for 15 min. 

The solution was then filtered through a glass filter labeled B2 without vacuum into a laboratory beaker. A 14 mL volume of the tested solution was added to the viscometer, which was placed in the thermostat at a temperature of 20 ± 1 °C. After 5 min, the solution was pushed through the viscometer twice with a pipette, then the measurement was performed. The solution was drawn over the upper mark, the pipette was removed, and the flow time between the upper and lower marks was measured. The flow time of pure Cuen was determined in the same way. The calculations were defined by Schultze–Blaschke [26,33]:

(1) $$[\eta ]=\frac{\eta_{sp}}{c(1+0.29\eta_{sp})}$$

where [η] is intrinsic viscosity, n_sp_ is specific viscosity, and c is the concentration, in g/mL, of cellulose in the Cuen solution.

(2) $$\eta_{\mathrm{sp}}=\frac{t-t_{0}}{t_{0}}$$

where n_sp_ is specific viscosity, t_0_ is flow time of Cuen, and t is the flow time of the solvent

(3) $$\bar{DP}=2.23\cdot [\eta ]$$

where DP the degree of polymerization and [η] is intrinsic viscosity

(4) $$\log\;\bar{DP}=1.32\cdot \log[\eta ]-0.51$$

(5) $${\bar{M}}_{\eta }=\bar{DP}\cdot M_{\mathrm{rcel}}$$

where ${\bar{M}}_{\eta }$ is number average molecular weight and M_rcel_ (C_6_H_10_0_5_) = 162 g/mol.

The degree of polymerization was used to determine whether the structure of the macromolecules of cotton compresses changes under the influence of different sterilizations.

### 2.5. Determination of Maximum Force and Elongation

**Figure 4:**
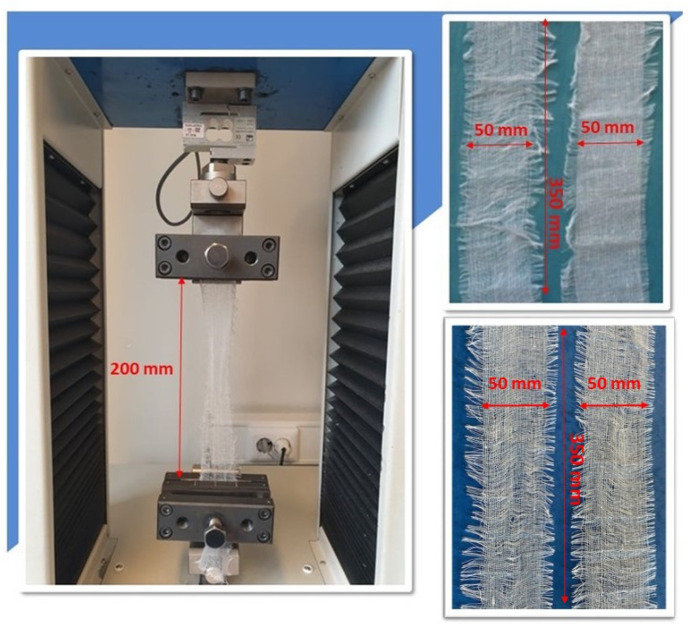
Appearance of the compress sample prepared for the test in the clamps of the dynamometer (top right—in dry condition, bottom right—in wet condition).

A standardized test of the breaking force and elongation of compresses was carried out. The test of breaking strength and elongation of compresses was carried out in accordance with the following standards EN 14079:2008 and EN ISO 13934-1:2008 [4,34]. The strength test of the compresses was carried out in dry and wet conditions. The test was carried out on a constant-rate-of-traverse strength machine Tensolab 3000, Mesdan S.p.A., Raffa, Italy (Figure 4).

The test samples were prepared in the form of strips measuring 350 × 60 mm (finished measuring width 50 mm), with 5 test samples in the warp direction and 5 test samples in the weft direction. The measured length of the test sample (distance between the clamps) was 200 mm. A load-controlled test was performed in which the load rate was controlled so that the load increase per unit time was constant (dP/dt = const). The speed of movement of the clamps was 100 mm ± 10 mm per minute. A consequence of such a loading regime is that the test is terminated when the peak load is reached, as the specimen cannot bear a higher load. In order to ensure the same measuring length for repeated measurements, the test sample was provided with a pretension that depended on the surface mass of the sample. The test results are given separately for warp and weft in the form of the following values: breaking force [N] and elongation at break [%] with the calculation of the basic statistical indicators. The test was carried out in a wet state, as cotton fibers are stronger when wet than when dry, unless they are chemically damaged [25]. The purpose of this test was to determine whether or not the cotton fibers had been damaged by the sterilization process. The results of the wet strength test were expressed in terms of the relative wet strength, fr [35]:

(6) $$f_{r}=\frac{F_{max\;}(wet\;condition)}{F_{max}(dry\;condition)}\cdot 100\;\left[\%\right]$$

If the material is damaged, the relative wet strength is less than 100%. The tests under the wet state were carried out using the same method, the same procedures and dynamometer as under the dry state. Immediately prior to exposure, the tested sample is immersed in distilled water to which a wetting agent has been added. The wet sample is clamped in the clamps of the force gauge and loaded under the prescribed conditions until it breaks. In order to obtain reproducible, i.e., clearly meaningful test results, the tests were carried out under strictly defined humidity and temperature conditions, i.e., in an air-conditioned laboratory for physical-mechanical tests of textiles, in an air space with a temperature of 20 ± 2 °C and a relative humidity of 65 ± 4% [36]. Testing the strength of compresses when wet is a suitable method for detecting damage, as textile materials exhibit a greater reduction in strength when wet. It was assumed that the testing of compresses and the results obtained could contribute to understanding the influence of different sterilizations on cotton compresses.

### 2.6. Determination of the Morphology of the Materials

The morphological properties of the compresses were examined using a scanning electron microscope (SEM) TESCAN, VEGA III EASYPROBE, Brno, Czech Republic. The SEM had an operating voltage of 20 kV and a secondary electron detector. The samples were coated with Au/Cr, and the magnifications were 2500× and 10,000×. The thickness of the Au/Cr coating was 1–20 nm, the working distance was 25 mm, and 10 randomly selected regions per sample were analyzed.

### 2.7. Methods of Testing Damage to Cotton Materials

**Figure 5:**
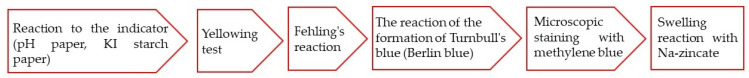
Protocol of the damage testing methods.

The type and possible intensity of damage to compresses caused by sterilization was determined by applying the following test procedures: reaction to the indicator (pH paper, KI starch paper), yellowing test, Fehling’s reaction, the reaction of the formation of Turnbull’s blue (Berlin blue), microscopic staining with methylene blue and swelling reaction with Na-zincate (Figure 5).

#### 2.7.1. Reaction to the Paper Indicator

To detect damage to the compresses by acid or alkali, moistened pH paper is placed in the fold of the tested material and a weight is applied from above. After 5 min, the color change is checked. If very small amounts of acid or alkali are present, it is best to prepare an aqueous extract and determine the pH value with a measuring device. Oxidizing agents are tested in the same way, except that KI starch paper is used, which takes on blue color under the influence of oxidizing agents. If it is not possible to detect the presence of chemicals on the material, then the changes are analyzed using various physical or chemical methods. The performance of reactions on paper indicators is fast; a color change is observed visually, which immediately indicates whether the sample has been damaged by acid or alkali.

#### 2.7.2. Test Yellowing

The compress sample is heated in a dryer at a temperature of 130–150 °C for several hours. The greater the damage, the greater the yellowing of the fibers. For comparison, it is best to insert a sample of an unsterilized compress too. The yellowing test serves as a quick test to determine whether the compress, i.e., the cotton fiber, is damaged. In order to obtain a more objective result, a numerical evaluation of the yellowing results is carried out by comparing the changes with a standard grey scale (color fastness test). In the color fastness test, the extent of the color change between the unsterilized sample and the sterilized sample after the test must be determined. The scale consists of five pairs of grey material numbered from 1 (poor; strong visual change) to 5 (good; no visual change) [37]. After treatment, the sample is compared with the original, unsterilized material, and any loss of color is assessed using the grey scale. The scale consists of pairs of grey color patches: one half of the pair is always the same shade of grey, and the other half becomes progressively lighter. The grey scale has 9 possible values, half a point in half a point, from 1 to 5 (1, 1–2, 2, 2–3, 3, 3–4, 4, 4–5, 5). The grey scale for evaluating the change in color was from SDC Enterprises Limited, Holmfirth, United Kingdom and corresponds to ISO 105-A02 [38].

#### 2.7.3. Fehling Reaction

Mix 10 mL of solution I with 10 mL of solution II, and heat to boiling (Table 1). Add the sample to the solution and boil for 3 min, then remove and rinse well.

The presence of red or pink colored stains indicates chemical damage. The coloration is caused by the presence of aldehyde groups in the damaged sample, which reduce alkaline copper salts and precipitate as insoluble red copper(I) oxide.

(7) $${\mathrm{Cu}}^{2+}\;\overset{reduction}{\to }\mathrm{Cu}↓{\mathrm{Cu}}_{2}O$$

#### 2.7.4. Turnbull Blue Staining Reaction

The carboxyl groups present in oxycellulose have the property of firmly binding heavy metal ions, especially in their bivalent form [39]. Based on this fact, a method for the qualitative detection of these groups in oxycellulose is used. The analyzed compress sample is treated for 10 min in a 1% FeSO_4_ solution at room temperature, then washed thoroughly in running water. It is then treated for 5 min in a 1% K_3_[Fe(CN)_6_] solution, also at room temperature. Then, it is rinsed again under running water. Parts of the sample that contain carboxyl groups due to damage turn an intense greenish-blue color. Undamaged cellulose colors poorly.

#### 2.7.5. Microscopic Staining with Methylene Blue

Finely chopped fibers of the potentially damaged specimen are placed on a slide and covered with a coverslip. Then, a drop of methylene blue solution is added to the edge of the slide. The preparation is left in the oven at 40 °C for 3 min. Before microscopy, the fibers are washed thoroughly with distilled water, which is added to one side of the slide, while on the other side, it is removed from the preparation using filter paper. The rinsing is carried out until the dye is removed. Undamaged fibers are not dyed, while chemically damaged fibers are dyed or remain undyed depending on the type of damage.

#### 2.7.6. Swelling Reaction with Zincate

Fibers that have been dyed with methylene blue are further processed with a Na-zincate solution. A drop of the solution is put on the edge of the coverslip, then filtered with a filter paper. It is necessary to be very patient and take time to ensure that the water present is completely washed out. This can be seen from the change of blue color to a light violet, and the fibers no longer move due to swelling. At the end of the swelling, the Na-zincate is washed away with water, giving the fibers back their blue color if they have been dyed. The reaction should be observed from the first contact of the fibers with the zincate until the end of washing. By adding zinc it can be observed that undamaged fibers unwind, becoming cylindrical, and the ends bulge like a mushroom; in fibers damaged by acid, if incisions appear in the first phase of swelling, the more severe the damage; fibers damaged by microorganisms (bacteria, molds) show cracks, cuts and segmentation [26].

The above-mentioned phenomena must be supplemented by observing the fibers during washing. In this phase, it is finally determined whether the fiber has been chemically or mechanically damaged. Changes during rinsing include the following:

- Fibers that are slightly chemically damaged swell during washing and then return to their original size;
- Chemically damaged fibers swell up considerably; it can be seen how the dissolved cellulose escapes and settles in the form of grains. Fibers damaged by acids show cuts that turn into transverse cracks;
- Normal or mechanically damaged fibers change little when washed, except that irregular tears can be observed in fibers damaged by friction [26].

### 2.8. FTIR Spectroscopic Characterization

FTIR characterization of compresses was performed using a Fourier transform infrared spectrometer (Spectrum 100 FTIR, Perkin Elmer, Waltham, MA, USA) using the attenuated total reflectance (ATR) technique. The spectra of the samples were recorded in a frequency range from 400 to 4000 cm^−1^ in diffuse reflectance mode with a resolution of 4 cm^−1^. All spectra at room temperature were recorded. 

### 2.9. Determination of Swelling Capacity Degree

The degree of swelling capacity was determined according to the British Pharmacopoeia, Annex XX L, A219 [40]. According to this method, first the sinking time and then the capacity were determined. After the basket containing the sample was removed from the water, it was allowed to drain for 30 s. The sample was then weighed to the nearest 10 mg. The weight of the wet samples was calculated, and then the procedure was repeated on two further samples. The average value was calculated. The degree of swelling (S) is calculated using the following formula:

(8) $$S=\frac{{W_{wet}-W}_{dry\;}}{W_{dry}}\cdot 100\;[\%]$$

## 3. Results and Discussion

The original dimensions of the compresses used were 20 × 10 cm, with 12 layers. The composition showed that the compresses were made of cotton fibres. The mass per unit area was determined to be 26 g m^−2^ (compress 1) and 25 g m^−2^ (compress 2). The thickness was determined to be 0.20 mm (compress 1 and compress 2). The compresses were opened, unpacked and suspended in a controlled atmosphere for 24 h immediately before the test.

The results include the pH of the water extract from wet-processed textiles, degree of polymerization, maximum force and elongation, morphology of the materials and methods of testing damage to cotton materials. Table 2 contains the codes of the samples used in this work.

### 3.1. Results of Activity of Hydrogen Ions

All tested compress samples, unsterilized and sterilized, have a neutral pH value (7) and are considered neither acidic nor alkaline. The pH value is a safety index for textiles, whereby a pH value of 7 does not affect the performance of the compresses themselves and does not cause inflammation of the body on contact with blood, tissue and exudates.

### 3.2. Determination of the Degree of Polymerization

A large number of repetitions of the DP determination were carried out. The results of the first measurements were discarded, and the results presented here are based on 10 measurements to determine the flow time for each sample of compresses. The average degree of polymerization of cotton varies between 9000 and 15,000, depending on the type of cotton used [41]. The type of cotton used to make the compresses was not indicated on the compresses tested, so it was not possible to say with certainty what type of cotton was used. The results of DP can be influenced by the quality of cotton raw materials in different batches, slight differences in the production process, genetic factors and growth forces determined by climate and growing conditions, and are a characteristic of natural fibers. It was stated that the compresses were bleached before sterilization. 

In the case of bleached cotton, the DP is between 1500 and 300 [3]. Our results for the DP of cotton were between 2131 and 2603 (Table 3). It can be assumed that the results were influenced by the sterilization methods. With a DP of more than 1500, it can be assumed that some kind of restructuring takes place in the macromolecule of the cotton. It is assumed that sterilization has promoted the close and correct alignment of the long cellulose molecules in parallel rows and that the glucose rings lie in the same plane as the oxygen that connects them in the cellulose. In such an alignment, hydrogen bonds can be formed between the hydroxyl groups of one chain and the hydroxyl groups of the other chain [42]. This leads to cross-linking of the macromolecules [25]. This cross-linking creates crystalline areas in cellulose fibers. In the structure of cellulose, there are also non-crystalline areas that are accessible to water molecules, coloring agents, etc. [43]. Physical-chemical processes can take place in these areas [25]. Due to the effect of the forces, the changes that are responsible for the mechanical deformation of the fibers initially take place in these areas [25].

The intrinsic viscosities of the individual samples are listed in Table 3. They are in the order [η] 1—U < [η] 1—SS/2—U/2—SS < [η] 1—ETO < [η] 2—ETO.

Intrinsic viscosity is the most important variable for describing the viscous behavior of a polymer solution. This is because the intrinsic viscosity indicates the true viscosity-increasing properties of a polymer independent of its concentration in the solution [44]. Intrinsic viscosity is not a direct measure of the polymer molecular weight, but a measure of the size of a polymer spiral in solution. The value of [η] increases with the interaction between the solvent and the polymer molecules [45]. The improvement in [η] is attributed to the increase in DP, which is visible in Table 4 for all samples.

### 3.3. Determination of Maximum Force and Elongation

The tensile strength of the samples was tested under dry and wet states. The maximum force, the elongation and the results of the *t*-test are listed in Table 4 and Table 5. A statistical *t*-test was performed to determine whether or not the sterilization process had an effect on the tensile strength of the cotton fibers. It was hypothesized that sterilization has no influence on the compresses and that there is no difference between sterilized and non-sterilized samples. The unsterilized sample (1—U) was statistically analyzed in relation to the steam-sterilized sample (1—SS), as was the unsterilized sample (1—U) in relation to the ethylene oxide sample (1—ETO). The same analogy of analyzing the samples with the *t*-test was maintained when analyzing the other samples. The *t*-test was carried out in both production directions (warp, weft). The results for the maximum force were subjected to the *t*-test, taking into account that the possible influence of sterilization reflects the results for the strength (reduction). In addition, the results of the wet strength test were expressed as relative wet strength, as shown in Figure 5.

The *t*-test was used to test the null hypothesis: sterilization did not affect the compresses; there is no difference between sterilized and non-sterilized samples. For all samples tested, the *p*-value was greater than 0.05, except for sample 1—ET0 (0.01) in weft direction, which was tested under the dry state. For all samples (except 1—ETO, dry state), the hypothesis was accepted, and the differences were within the limits of random deviations, i.e., they were not statistically significant. For sample 1—ETO, the hypothesis was rejected, and the differences were therefore significant. Since the hypothesis was only rejected for one sample, it would be necessary to repeat the measurements and increase the number of measurements in order to reach a conclusion rejecting the hypothesis. The result could be influenced by the preparation of the compresses, which leads to a shift in the density of the warp and weft threads and consequently affects the results of the tensile strength and elongation at break of the cotton compresses. The results listed in Table 4 and Table 5 show that the unsterilized compresses had slightly higher values for breaking strength and elongation at break in the dry state compared to the sterilized compresses. In the wet state, an increase was observed during steam sterilization, which can be interpreted as a fundamental property of cotton, namely, that its strength increases in the wet state, e.g., under increased moist conditions such as steam sterilization.

**Figure 6:**
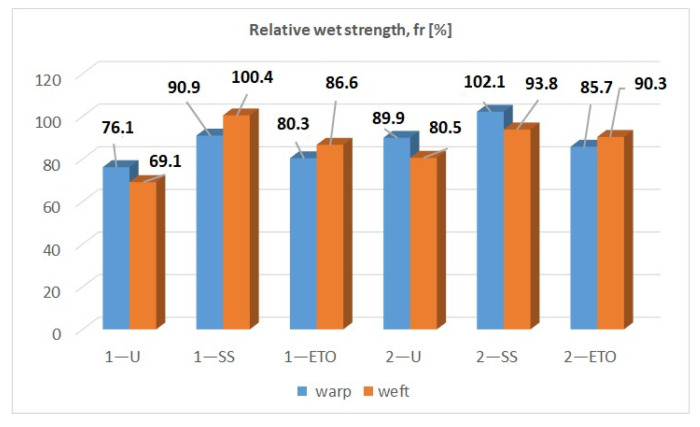
Results for relative strength in wet state.

The results of the wet strength test, expressed as relative wet strength, fr, are shown in Figure 6. It is known from theory that the relative wet strength is less than 100% when the material is damaged [35]. Sample 1—SS in weft direction (100.4%) and sample 2—SS in warp direction (102.1%) have values above 100%. According to the theory, the samples are not damaged. The values for all other samples are below 100%. Figure 6 shows that the untreated samples have lower values than the sterilized samples. It follows that the untreated samples may be affected by bleaching. It is assumed that the preparatory processes on the compresses prior to sterilization influence the lower values of the relative wet strength. The results in the warp direction for sample 1, for example, are 1—U (76.1%), 1—SS (90.9%) and 1—ETO (80.3%). The other samples show the same analogy, i.e., relative strength values are untreated < steam sterilization > ethylene oxide sterilization.

### 3.4. Determination of the Morphology of the Materials

Table 6 shows the scanning electron images of the test compress samples. Samples 1—U and 2—U represent unsterilized compresses, while samples 1—SS and 2—SS were treated by steam sterilization, and samples 1—ETO and 2—ETO by ethylene oxide sterilization.

The scanning electron images suggest that the morphological properties of the surface of the examined compresses have not changed as a result of sterilization, as no significant changes can be seen between the samples. At a magnification of 10,000×, a slight change in the form of a crack can be seen on sample 1—SS, which is not necessarily a result of sterilization, but could have been due to damage during sample preparation. Dots appear on samples 1—ETO and 2—ETO, which are thought to be residues of the precipitant, as these samples were sterilized with ethylene oxide, but we cannot say for sure, as we do not know the sterilization conditions, which are trade secrets of the manufacturer. The compresses sterilized with ethylene oxide were tested in accordance with ISO 10993-7. The standard provides information on the potential hazards of ethylene oxide residues and by-products that may remain on the compresses after sterilization. ISO 10993-7 is crucial for user safety, especially because it ensures that compresses are free from harmful ethylene oxide residues, which is critical for the safety of patients, healthcare professionals and other users [46,47]. Based on knowledge of this standard, it can be concluded that the dots are both acceptable and indicate non-hazardous levels of residue, or that the sample was contaminated during SEM imaging.

### 3.5. Methods of Testing Damage to Cotton Materials

Section 3.5.1, Section 3.5.2, Section 3.5.3, Section 3.5.4 and Section 3.5.5 present the results of various test methods to determine the possible intensity of damage to compresses caused by sterilization. It is characteristic of cotton and therefore also of cotton compresses that physical-chemical changes can occur in the cellulose on contact with various chemical agents. The reactions of the cellulose vary depending on the types of reagents and the conditions under which they act. By using a variety of methods to test the damage to the cotton material, it is clear that exposure to alkali did not lead to alkalization of the cellulose, i.e., alkaline cellulose, nor did exposure to acidic reagents lead to damage and the formation of hydrocellulose, i.e., oxycellulose, as the length of the macromolecules increased (DP). It can be concluded that the sterilizations were carried out with care and caution and that no damage to the cellulose, i.e., the cotton compresses, was observed.

#### 3.5.1. Reaction to the Paper Indicator

Unsterilized and sterilized compresses have a neutral pH value (7) for the paper indicator. The pH value of the paper indicator was 7 for all samples tested. The results obtained are consistent with the results for hydrogen ion activity and confirm that the samples tested did not affect the performance of the compresses themselves and should not cause inflammation of the body when in contact with blood, tissue and exudates. Unsterilized and sterilized compresses tested with KI starch paper showed no change in color. It can therefore be concluded that no oxidizing agents were present in the compress samples tested. 

#### 3.5.2. Test Yellowing

**Figure 7:**
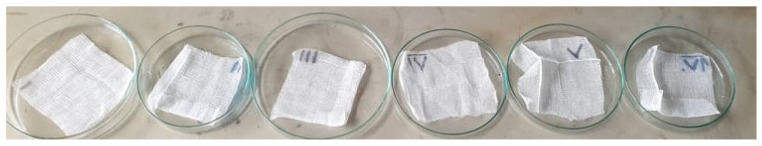
The appearance of the tested compresses after the yellowing test.

After the compresses had been subjected to the yellowing test, no change in the color of the tested compresses was visually discernible. There were no yellowish tones. It can be therefore assumed that the material was not damaged, which prompted us to carry out further tests with the Fehling reaction. Figure 7 shows the samples after the yellowing test.

The grey scale was used to visually assess the color fastness of compresses. The grey scale for evaluating color changes has five levels. Grade 5 means no yellowing and indicates unchanged coloring, while grade 1 indicates very weak color fastness, i.e., strong yellowing. The samples tested showed no color change, i.e., there was no yellowing.

#### 3.5.3. Fehling Reaction

**Figure 8:**
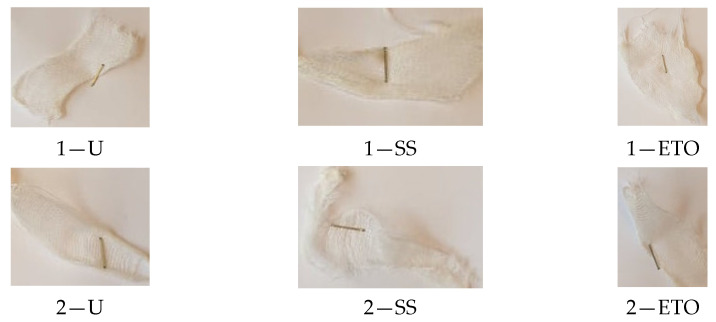
Appearance of the samples after the test.

After testing the compresses with the Fehling reaction, it was obvious that no stains formed (Figure 8). It can therefore be concluded that there were no aldehyde groups present that could indicate damage to the compresses. It was necessary to carry out further tests with Turnbull blue.

#### 3.5.4. Turnbull Blue Staining Reaction

**Figure 9:**
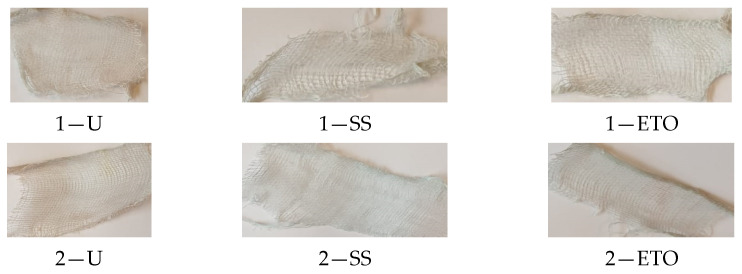
Appearance of the samples after the test.

After treatment of the compresses with Turnbull’s blue, a barely perceptible change in the color of the specimens could be observed at the edges of the sample (Figure 9). This change could indicate contamination, i.e., damage to the compresses during preparation of the samples for the test.

#### 3.5.5. Microscopic Staining with Methylene Blue and Swelling Reaction with Na-Zincate

**Figure 10:**
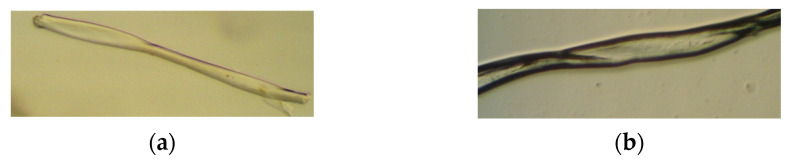
Microscopic (**a**) staining with methylene blue and (**b**) swelling with Na-zincate.

Microscopy of the samples with methylene blue revealed that the fibers did not change color, as can be seen in Figure 10. Since the color did not change, it can be assumed that the cotton fibers of the compresses were not damaged during sterilization. Microscopy and observation of the swelling of the cotton fibers of the compresses with Na-zincate showed no cracks or cuts on the fibers, and it is assumed that the cotton fibers were not damaged during sterilization.

### 3.6. Results of the FTIR Spectroscopic Characterization

**Figure 11:**
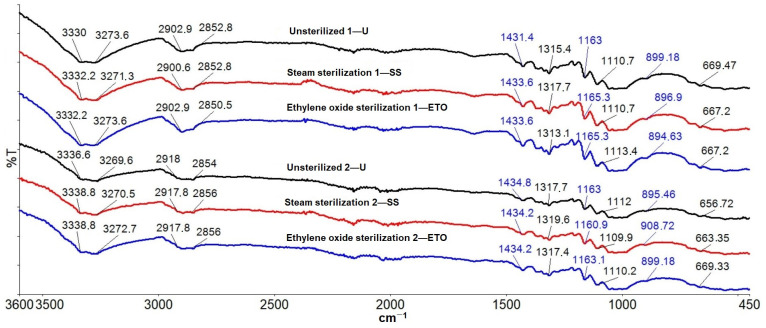
FTIR spectra of compresses.

Figure 11 shows the ATR-FTIR analyses of samples before and after both sterilization treatments.

FTIR-ATR spectroscopy was used as a technique to determine whether sterilization has an influence on spectral changes, i.e., affects the bands of the cotton. The spectra of the samples were recorded in a frequency range from 400 to 4000 cm^−1^, but they are shown from 400 to 3800 cm^−1^ to give the best possible insight into all bands. Typical bands of cellulose, lignin and hemicellulose were observed in the ATR-FTIR spectrum of the unsterilized and sterilized cotton compresses. The assignments of the bands of the cotton fiber were taken from the literature: OH stretching at ~3333–3286 cm^−1^; C-H symmetric stretching at ~2897 cm^−1^; HCH and OCH in-plane bending vibration at ~1428 cm^−1^; in-plane bending at ~1363 cm^−1^; CH_2_ vibration at C_6_ ~1314 cm^−1^; C-O-C symmetric stretching, OH in-plane deformation at ~1205 cm^−1^; C-O-C asymmetric stretching at ~1160 cm^−1^; C-C, C-OH, C-H ring and side group vibrations at ~1054–983 cm^−1^; COC, CCO and CCH deformation and stretching at ~897 cm^−1^; and C-OH out-of-plane bending at ~662 cm^−1^ [48,49,50,51,52,53,54,55]. The peaks observed at 3330 cm^−1^ (1—U) and 3333.2 cm^−1^ (1—SS and 1—ETO) are characteristic of the hydroxyl groups (OH) of cellulose, lignin and water. The peaks at 2852.8 cm^−1^ (1—U and 1—SS) and 2850.5 cm^−1^ (1—ETO) are characteristic of the stretching vibration of C-H in cellulose and hemicellulose. The peaks at 1431.4 cm^−1^ (1—U) and 1433.6 cm^−1^ (1—SS and 1—ETO) are associated with the symmetric CH_2_ bending of cellulose. The peaks at 1315.4 cm^−1^ (1—U) 1317.7 cm^−1^ (1—SS) and 1313.1 cm^−1^ (1—ETO) belong to the bending vibrations of the C-H and C-O groups of the aromatic rings in cellulose polysaccharides. The peaks in the range 1200–800 cm^−1^ are attributed to the C–O and C–C stretching vibrations. The peaks at 899.18 cm^−1^ (1—U), 896.9 cm^−1^ (1—SS) and 894.63 cm^−1^ (1—ETO) indicate the presence of β-glycoside bonds between monosaccharides. Figure 11 shows the agreement with the peaks of unsterilized, steam-sterilized and ethylene oxide-sterilized cotton compresses. Each peak matches the peak of the two sterilization methods. All peak values correspond to the previously theoretically determined wave numbers. The peaks of the unsterilized (2—U) and sterilized (2—SS, 2—ETO) samples show the same analogous behavior as in sample 1 and coincide with all peaks of the unsterilized and the two sterilization methods (sample 2). It can be concluded that the results of the FTIR-ATR spectroscopic characterization confirm that there was no alteration of the characteristic bands of the cotton fiber by the sterilization processes.

### 3.7. Results for the Swelling Capacity Degree

Table 7 shows the result for the swelling capacity degree. According to the method, the immersion time was performed at the beginning of the measurement, with a time of 2 s for all tested samples listed in the table. 

The results of the determination of the swelling capacity were influenced by the sorption of the liquid by the cotton fibers themselves and the sorption in the spaces between the fibers, where the liquid is held by capillary forces [56]. The sorption of the liquid by the cotton fibers depends on the physical and chemical structure of the cotton fibers. Sorption in the inter-fiber spaces, on the other hand, depends on physical properties such as the wettability of the fibers, the surface energy of the fibers and the fiber geometry [56]. All tested samples showed a high water absorption capacity (values from 639.4 to 790.3%). Non-sterilized samples had the highest absorption capacity, followed by samples sterilized with ethylene oxide, and then samples sterilized by steam sterilization. It is evident that sterilization has an influence on the reduction of water absorption capacity. In the case of sample 2, for example, the unsterilized sample had an absorption capacity of 746.64%, and the sample sterilized with ethylene oxide, 743.0%. As the steam-sterilized samples were exposed to steam, it can be assumed that swelling occurred during the sterilization itself, and then again during the capacity test, which then reduced the re-swelling capacity. The values obtained were slightly lower (e.g., 712.37% -1—SS) than those of the original (e.g., 755.47% -1—U), but still high capacity values that were sufficient for the intended purpose.

## 4. Conclusions

The aim of this work was to determine the changes in the chemical and physical-mechanical properties of gauze compresses under the influence of various sterilizations (steam sterilization and sterilization with ethylene oxide). The methods for testing possible damage to cotton materials (pH value (pH paper, KI starch paper), yellowing test, Fehling reaction, reaction to the formation of Turnbull blue (Berlin blue), microscopic staining with methylene blue and swelling reaction with Na-zincate) did not show that the sterilizations affected the cotton compresses. The scanning electron images showed that the morphological properties of the surface of the examined compresses had not changed because of the sterilization, as no significant changes between the samples could be detected. The results of the FTIR-ATR spectroscopic characterization confirmed that there was no change in the characteristic bands of the cotton fiber due to the sterilization processes. However, the results of the determination of the degree of polymerization showed that the DP of cotton was between 2131 and 2603, while in our case of bleached cotton, the DP of cotton must have been between 1500 and 300. It can be assumed that sterilization processes promote the close and correct alignment of the long cellulose molecules in parallel rows and that the glucose rings lie in the same plane as the oxygen that connects them in the cellulose. Hydrogen bonds can be formed between the hydroxyl groups of one chain and the hydroxyl groups of the other chain, which leads to cross-linking of the macromolecules and creates crystalline areas in the cellulose fibers. There are also non-crystalline areas in the structure of cellulose that are accessible to water molecules, dyes, etc., so that corresponding physical-chemical processes can take place in these areas. Due to the effect of the forces, the changes that are responsible for the mechanical deformation of the fibers initially take place in these areas. This was followed by the results of the wet strength test. The results of the wet strength test, expressed as relative wet strength, showed values below 100%. The samples had values below 100%, except for sample 1—SS in the weft direction (100.4%) and sample 2—SS in the warp direction (102.1%). It is known from theory that the relative wet strength is less than 100% when the material is damaged. On this basis, sterilization processes influence the compresses and the results presented analogously: values of relative strength of untreated < values of relative strength of steam sterilization > values of relative strength of ethylene oxide sterilization. The *t*-test performed on the strength results showed that the *p*-value was greater than 0.05 for all samples tested, except for sample 1—ET0 (0.01) in the weft direction, which was tested in the dry state. For all samples (except 1—ETO, dry state), the hypothesis was accepted, and the differences were within the limits of random deviations, i.e., they were not statistically significant. For sample 1—ETO, the hypothesis was rejected, and the differences were therefore significant. As the hypothesis was only rejected for one sample, the measurements would have to be repeated, and the number of measurements increased. The result could be influenced by the preparation of the compresses, which leads to a shift in the density of the warp and weft threads, which consequently affects the results of the tensile strength and elongation at break of the cotton compresses. The degree of swelling capacity showed that the non-sterilized samples had the highest capacity, followed by the samples sterilized with ethylene oxide and the samples sterilized with steam. As the steam-sterilized samples were exposed to saturated steam under pressure, it can be assumed that swelling occurred during the sterilization itself, and then again during the capacity test, which then reduced the re-swelling capacity. All samples tested showed a high water absorption capacity (values of 639.4 to 790.3%). The results obtained are a contribution to the innovation of the topic of this work and a scientific confirmation for manufacturers and all those interested in the influence of the sterilization process on natural fibers (cotton).

## Figures and Tables

**Table 1:** Fehling reagents (solution I and solution II) [26].

Solution I	Solution II
69.28 g/L CuSO_4_ crystalline	346 g/L K—Na tartrate
	100 g/L NaOH

**Table 2:** Sample codes.

Compress Batch Number	1			2		
Sample Code	1—U	1—SS	1—ETO	2—U	2—SS	2—ETO
Treatment	unsterilized	steam sterilization	ethylene oxide sterilization	unsterilized	steam sterilization	ethylene oxide sterilization

**Table 3:** Results for the degree of polymerization.

Sample Code	Cuen	1—U	1—SS	1—ETO	2—U	2—SS	2—ETO
The flow time [s]	128	183	194	196	195	195	197
η_sp_	/	0.44	0.53	0.54	0.53	0.53	0.55
η	/	975.52	1148.48	1167.21	1148.48	1148.48	1185.86
DP	/	2131	2519	2561	2519	2519	2603
*M_n_* [g mol^−1^]	/	352,416.33	414,899.89	421,666.28	414,899.82	414,899.82	428,403.78

η_sp_ is the specific viscosity, η is the intrinsic viscosity, DP is the degree of polymerization, *M_n_* is the average molecular weight, and Cuen is copper-ethylene diamine hydroxide.

**Table 4:** The strength of the tested samples in the dry state.

Sample		1			2		
Sample Code		1—U	1—SS	1—ETO	2—U	2—SS	2—ETO
warp	n	F [N]	F [N]	F [N]	F [N]	F [N]	F [N]
	1	93.4	65.9	72.4	94.4	82.2	86.6
	2	87.1	74.4	88.7	94.5	61.4	94.6
	3	84.0	87.4	83.8	94.4	87.8	94.6
	4	77.9	79.4	76.1	94.4	86.5	94.5
	5	88.9	70.3	74.4	94.4	94.6	87.1
	X	86.3	75.5	79.08	94.42	82.50	91.46
	σ	5.8	8.33	6.90	0.05	12.62	4.21
	V [%]	6.66	11.04	8.72	0.05	15.30	4.61
*t*-test	t-stat	/	1.83	1.61	/	2.11	1.56
	t-crit	/	2.78	2.78	/	2.78	2.77
	df	/	4	4	/	4	4
	p	/	0.14	0.18	/	0.10	0.19
	Decision	/	+	+	/	+	+
weft	1	46.2	49.2	38.5	52.7	48.2	41.7
	2	53.5	46.6	41.3	46.5	45.6	45.0
	3	50.2	39.3	43.3	48.97	43.8	53.6
	4	49.8	55.7	41.1	46.2	48.3	50.95
	5	61.5	46.5	40.7	51.6	40.8	44.6
	X	52.5	47.4	40.97	49.2	45.4	47.2
	σ	5.76	5.90	1.70	2.94	48.22	41.76
	V [%]	11.04	12.44	4.16	5.97	45.64	45.03
*t*-test	t-stat	/	1.19	4.41	/	1.77	0.65
	t-crit	/	2.78	2.13	/	2.78	2.78
	df	/	4	4	/	4	4
	p	/	0.30	0.01	/	0.15	0.65
	Decision	/	+	-	/	+	+

*t*-test = student *t*-test; df = the degrees of freedom for the *t*-test; t-stat = the test statistic t; p = value for a two-tailed *t*-test; t—critical = two-tailed t-critical test; decision = acceptance (+) or rejection (−) of the Hypothesis: Sterilization has no effect on the compresses, and there is no difference between sterilized and non-sterilized samples.

**Table 5:** The strength of the tested samples in the wet state.

Sample		1			2		
Sample Code		1—U	1—SS	1—ETO	2—U	2—SS	2—ETO
warp	n	F [N]	F [N]	F [N]	F [N]	F [N]	F [N]
	1	58.6	64.9	58.3	80.2	84.3	99.1
	2	70.1	70.3	68.5	78.2	90.5	78.0
	3	67.0	68.8	59.4	92.4	81.4	68.5
	4	63.6	71.1	65.3	93.4	90.7	78.6
	5	69.3	68.1	66.1	80.3	74.2	68.0
	X	65.7	68.6	63.5	84.9	84.2	78.4
	σ	4.7	2.4	4.4	7.3	6.9	12.6
	V [%]	7.15	3.50	6.98	8.65	8.17	16.11
*t*-test	t-stat	/	−1.71	1.40	/	0.17	0.88
	t-crit	/	2.78	2.78	/	2.78	2.75
	df	/	4	4	/	4	4
	p	/	0.16	0.23	/	0.86	0.43
	Decision	/	+	+	/	+	+
weft	1	31.5	53.7	38.0	34.9	43.1	35.7
	2	42.6	45.7	32.5	36.2	43.2	44.4
	3	43.4	41.6	31.5	37.8	41.5	36.5
	4	30.4	50.1	29.7	47.2	42.6	50.3
	5	33.4	47.1	46.0	42.2	42.5	46.1
	X	36.3	47.6	35.5	39.6	42.6	42.6
	σ	6.3	4.6	6.6	5.0	0.7	6.3
	V [%]	17.25	9.63	18.62	12.67	1.66	14.85
*t*-test	t-stat	/	−1.71	0.15	/	−1.25	−1.84
	t-crit	/	2.78	2.78	/	2.78	2.78
	df	/	4	4	/	4	4
	p	/	0.07	0.89	/	0.28	0.14
	Decision	/	+	+	/	+	+

*t*-test = student *t*-test; df = the degrees of freedom for the *t*-test; t-stat = the test statistic t; p = value for a two-tailed *t*-test; t—critical = two-tailed t-critical test; decision = acceptance or rejection of the hypothesis (sterilization did not affect the compresses (cotton fibers)).

**Table 6:** Morphology of the cotton fiber of compresses.

Sample Code/ Magnification	Morphology of the Materials	
	2500×	10,000×
1—U	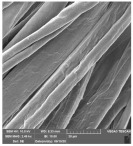	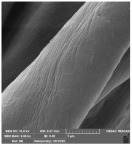
1—SS	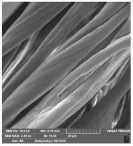	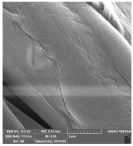
1—ETO	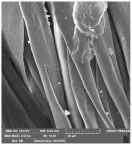	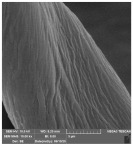
2—U	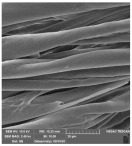	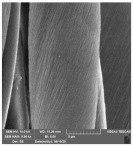
2—SS	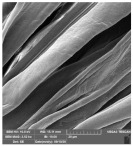	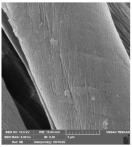
2—ETO	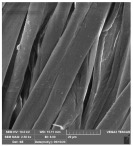	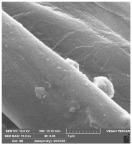

**Table 7:** Results for the swelling capacity degree.

Sample Code			1—U	1—SS	1—ETO	2—U	2—SS	2—ETO
n	1	W_dry_ [g]	0.9918	0.9933	1.0009	0.9983	1.0002	1.0067
		W_wet_ [g]	8.7762	8.2827	8.8496	8.6432	7.5265	8.9627
		S [%]	784.9	733.9	784.2	765.8	652.5	790.3
	2	W_dry_ [g]	0.9719	0.9920	0.9931	0.9817	1.0024	0.9960
		W_wet_ [g]	8.6512	7.9312	8.0609	8.6479	8.3025	7.9065
		S [%]	790.1	699.5	711.7	780.9	728.3	693.8
	3	W_dry_ [g]	0.9777	1.0046	0.9810	0.9801	0.9874	0.9516
		W_wet_ [g]	7.7373	8.0743	8.0054	7.7746	7.3004	8.0401
		S [%]	691.4	703.7	716.0	693.2	639.4	744.9
X			755.47	712.37	737.30	746.64	673.4	743.0
σ			45.352	15.323	33.210	38.283	39.187	39.419
V [%]			6.00	2.15	4.50	5.13	5.82	5.31

W_dry_ is weight of dry sample, W_wet_ is weight of wet sample; m_basket_ used for the test was 2.9973 g; basic statistical indicators (X, σ, V) were calculated for the values of S of each analyzed sample.

## Data Availability

The original contributions presented in the study are included in the article; further inquiries can be directed to the corresponding author.
